# Practice guidelines in the context of primary care, learning and usability in the physicians’ decision-making process – a qualitative study

**DOI:** 10.1186/1471-2296-15-141

**Published:** 2014-08-20

**Authors:** Maria Ingemansson, Pia Bastholm-Rahmner, Anna Kiessling

**Affiliations:** 1 Department of Women and Childrens’ Health, Karolinska Institutet, Stockholm, Sweden; 2 Department of Learning informatics, Management and Ethics. Medical Management Centre (MMC), Karolinska Institutet, Stockholm, Sweden; 3 Department of Clinical Sciences, Karolinska Institutet, Stockholm, Sweden

**Keywords:** General practitioners, Decision-making, Diagnostic errors, Practice guidelines, Context, Learning, Usability

## Abstract

**Background:**

Decision-making is central for general practitioners (GP). Practice guidelines are important tools in this process but implementation of them in the complex context of primary care is a challenge. The purpose of this study was to explore how GPs approach, learn from and use practice guidelines in their day-to-day decision-making process in primary care.

**Method:**

A qualitative approach using focus-group interviews was chosen in order to provide in-depth information. The participants were 22 GPs with a median of seven years of experience in primary care, representing seven primary healthcare centres in Stockholm, Sweden in 2011. The interviews focused on how the GPs use guidelines in their decision-making, factors that influence their decision how to approach these guidelines, and how they could encourage the learning process in routine practice.

Data were analysed by qualitative content analysis. Meaning units were condensed and grouped in categories. After interpreting the content in the categories, themes were created.

**Results:**

Three themes were conceptualized. The first theme emphasized *to use guidelines by interactive contextualized dialogues.* The categories underpinning this theme: 1. Feedback by peer-learning 2. Feedback by collaboration, mutual learning, and equality between specialties, identified important ways to achieve this learning dialogue. Confidence was central in the second theme, *learning that establishes confidence to provide high quality care*. Three aspects of confidence were identified in the categories of this theme: 1. Confidence by confirmation, 2. Confidence by reliability and 3. Confidence by evaluation of own results. In the third theme, *learning by use of relevant evidence in the decision-making process*, we identified two categories: 1. Design and lay-out visualizing the evidence 2. Accessibility adapted to the clinical decision-making process as prerequisites for using the practice guidelines.

**Conclusions:**

Decision-making in primary care is a dual process that involves use of intuitive and analytic thinking in a balanced way in order to provide high quality care. Key aspects of effective learning in this clinical decision-making process were: contextualized dialogue, which was based on the GPs’ own experiences, feedback on own results and easy access to short guidelines perceived as trustworthy.

## Background

Physicians need to update their medical knowledge continuously. Practice guidelines, usually regularly updated, are intended to facilitate this. They are mainly provided as diagnosis-and evidence-based information available on the internet but also as printed material e.g. in brochures or books. Lectures or discussions with colleagues are other ways to communicate the content of practice guidelines. However, the implementation of practice guidelines in primary care is a complex task [1,2]. General practitioners (GPs) are expected to have the overall responsibility for complex patient cases with multiple health problems. Still, the available guidelines are not designed to guide the comprehensive treatment of patients with multiple diseases but rather to guide treatment of separate diagnoses. It is therefore not surprising that previous research has shown that there is a considerable gap between what is carried out by physicians in clinical practice and what should be carried out to achieve the care and target levels stated in the guidelines [1-5].

In the Swedish healthcare system a common directive in most practice guidelines is that relevant basal investigations should be performed and evaluated in primary care before the patients are referred to secondary care. There are high expectations of medical knowledge in many areas since the formal requirements to become a specialist in family medicine, e.g. a GP, in Sweden are equivalent as regard years in education and competence level to the requirements for any specialties in secondary care. However, the Swedish system to support continuous professional development (CPD) for physicians lacks formal requirements. Specialists, including GPs, are expected to keep themselves updated and to follow practice guidelines. Quality registers at local and national level are used in many disciplines to monitor the adherence to guidelines, and national comparisons aim to support increased adherence to the guidelines.

It is of great importance that CPD activities for GPs are based on principles of adult learning, and educational research indicates that methods which include interactive learning have the potential to increase knowledge and skills, and can change practice behaviour [6]. To get an effective and useful CPD in the complex context of primary care is a special challenge. Several qualitative studies have elucidated broad issues such as “GPs views on use of guidelines” or “Attitudes towards evidence-based medicine in general” [7,8]. Issues like these have a tendency to elicit general statements rather than provide deep understanding of facilitating or hindering factors regarding the use of guidelines in routine care. In contrast, to focus on specific diseases might narrow the perspective too much and impair comparison between implementation strategies and/or clinical conditions. Further, practice guidelines per se are not supporting interactivity. They are mostly formed as written texts which physicians are supposed to read, learn from and use in their daily practice. It is of outmost importance that development of guidelines and other knowledge sources is based on a deep understanding of the influence of the context and the interaction between healthcare professionals in learning and performance. As far as we know, research is lacking on how GPs contextualize, interact, learn from and use practice guidelines in their day-to-day decision-making process.

The aim of the study was to explore how GPs approach, learn from and use practice guidelines in their day-to-day decision-making process in the primary care context.

## Method

### Study design

A qualitative approach with focus group interviews was chosen. Focus group interviews have proven to be a useful method to provide in-depth information and to explore thoughts and feelings underlying behaviour [9,10].

The Dual Process Theory was used to interpret and discuss the conceptualized themes. The theory is a dominant model for understanding the complex process that underlies human decision-making [11-13].

The Dual Process Theory includes an intuitive (system 1) and an analytical (system 2) process. The theory is based on pattern recognition and the dual process this induces. If a patient presents with an illness that is recognized by the decision-maker e.g. a GP, the system 1 process will be engaged, which is fast but vulnerable to bias. If a patient presents with an illness that is not recognised by the decision-maker, the slower, more reliable system 2 process will be engaged, which requires more cognitive effort. There is a varying degree of interaction between system 1 and system 2 processes and the calibration between them depends on type of illness, the context and the experience of the decision-maker. The repeated appearance of similar situations using the system 2 process leads to a pattern recognition and relegation to the system 1 process. However, either system may override the other. A system 2 process override may result in decision-making characterized by control or surveillance. A system 1 process override may on the other hand result in a decision-making process where practice guidelines are overridden in favour of individual clinical judgment. There is tendency in the Dual Process Theory system to strive for a state requiring the least cognitive effort, the “cognitive miser” function.

The study followed the principles outlined in the Declaration of Helsinki, 1964 and was approved by the Regional Ethical Review Board in Stockholm, Sweden, Diary number: 2011/1071-32. Prior to the interviews all participants were informed both in writing and verbally about the study and that confidentiality was ensured. Written informed consent was obtained from all the participants.

### Setting and sample

The GPs in this study were selected from 14 primary healthcare centres (PHCs) in the north-western part of Stockholm, Sweden in 2011. The 14 PHCs had earlier shown interest in developing practice guidelines. All the physicians that worked at the 14 PHCs during the study time, in total 132 (85 women), were invited to participate in the focus group interviews by e- mail, with two reminders.

Twenty-two GPs (16 women), representing seven of the PHCs agreed to participate in the study. The GPs had a median of seven years of experience as physicians in primary care (interquartile range 3-14 years). By definition, GPs are specialists in family medicine. In this study, for simplicity reasons, we included all physicians at the PHCs willing to participate in the definition of GPs. The definition thus included specialists as well as residents in family medicine. In total, 16 of the GPs were specialists in family medicine while six were doing their residency. The seven PHCs that were represented employed between 6-19 GPs. One of the PHCs had a vacancy; the other six were fully staffed regarding physicians.

All the PHCs had access to written guidelines, scientific literature and professional journals e.g. printed as well as internet-based material.

### Data collection

In total, four focus group interviews were performed. Two interviews took place at the participants’ workplace while the other two interviews were performed in a conference room at the regional hospital. In the interviews performed outside the PHCs, only specialists participated; there were no residents (Table 1). The GPs that participated in the interviews performed at the PHCs were familiar with each other while the GPs participating in the other two interviews were not.

**Table 1:** Characteristics of the focus group interviews

**Focus group**	**Number of GPs participating**	**Number of PHCs represented**	**Setting**
1	4	4	Outside the PHC
2	8	1	At the PHC
3	7	1	At the PHC
4	3	3*	Outside the PHC

*Two of the PHCs were also represented at focus group interview number 1.

GP denotes General Practitioner. PHC denotes Primary Healthcare Centre.

Each interview lasted 1.5-2 hours. In three of the interviews (the two outside the PHCs and one performed at a PHC) there were lively discussions while the group dynamics were low in the last interview. One of the authors (MI), known to most of the participants through an earlier study and as a consulting specialist, conducted all the interviews. The second author (PBR), not known to the participants, acted as an observer, handled the tape-recorder and took field notes. Before the interview started, each GP answered a short questionnaire regarding their professional background, their use and access to guidelines and data regarding the number of GPs employed, and if there were any vacancies at their PHC. The results from the questionnaire are presented above. There were no incentives for the GPs to participate.

The interview guide consisted of an opening question, an introduction question, three core questions covering three important domains, and finally a closing question. The domains focused on how the GPs use practice guidelines in their decision-making, and factors that influence their decision how to approach a specific type of guideline. Furthermore, the interviews aimed to explore if and how the guidelines could encourage the learning process in the daily routine practice. The interview ended with a closing question where the participants were asked if they wanted to add something. This was done in order not to miss any data.

The interviews were semi-structured with open-ended questions [14]. Follow-up questions were used for clarification. The interview guide was tested in the first pilot focus group interview. Since it worked out well, the pilot focus group interview was included in the final study.

### Data analysis

Qualitative content analysis was performed [15,16]. The text was read several times to get a sense of the whole. The analysis started by identifying meaning units in the transcripts. The meaning units were then preliminarily sorted by content and meaning. In a second step, meaning units were condensed and labelled with codes while still preserving the meaning of the text. Codes that belonged together were grouped to form categories. In the next step the latent analysis was performed by analysing the content in the categories in a more abstract and interpretative way, and thus themes were created. The process of analysis is shown in Table 2.

**Table 2:** Process of analysis, with examples of meaning units, codes and categories corresponding to the themes

**Meaning unit**	**Codes**	**Category**	**Theme**
*”… in a very structured way introduced in our PHC. .. What should I do when I have diagnosed a patient with hypertension? … We circulated and then everybody got to talk… There was suddenly time for reflection.” - Female*	Structured group meetings give stimulation, opportunity for reflection	- Feedback by peer-learning	Learning to use guidelines by interactive contextualized dialogues.
*”When the guidelines were to be updated all the GPs were invited to a meeting…It was a short presentation followed by a long dialogue…” –Female*	Cooperation between primary and secondary care regarding new guidelines.	- Feedback by collaboration, mutual learning and equality between specialties	
“*After a couple of years I felt that … I know how to do this and continued to work without checking the guidelines… suddenly something went wrong… I got scared and started to check again.” – Male*	Confirmation of knowledge assures quality of care.	- Confidence by confirmation	Learning that establishes confidence to provide high quality care.
*” “I used to work in Gotland so I usually call the hospital there since I know everyone… I am always familiar with the person that I am talking to…” -”Female*	Reliability from consulting familiar, competent colleagues.	- Confidence by reliability	
*” I would like to see results…I want follow-ups, personal follow-ups…If I never get feedback on what I am doing…why should I care??” – Female*	Evaluation of improvement encourages adherence to guidelines.	- Confidence by evaluation of own results	
*“It should not be too compact…arranged in a recognizable pattern so the information is easy to find.” -Female*	Pedagogic lay-out	- Design and layout visualizing the evidence	Learning by use of relevant evidence in the decision-making process.
*”The biggest problem is lack of time…Oh God there is so much information, where should I start.” - Female*	Poor search- function time-consuming	- Accessibility adapted to the clinical decision-making process	

### Rigour

We chose the focus group interview method since interactions in a group of people with similar background on a specific topic enhance data quality [17]. To improve the judgment of transferability, the sample setting, data collection and analysis process were carefully described. The findings might therefore be transferrable to similar groups of GPs. In the fourth interview, much of the information had already been obtained in the earlier interviews, thus saturation was assumed. All the interviews were performed within seven months, which should contribute to trustworthiness by decreasing the risk of inconsistency [18]. The interviews were recorded, transcribed verbatim by one of the authors (MI) and checked against the audio-recording for accuracy. In order to ensure credibility, all the authors, who had different backgrounds and perspectives, performed the content analysis process and discussed categories and themes until consensus was obtained.

Quotations from the original interviews were selected in order to illustrate the results and to improve the credibility of the study.

## Results

Three themes were conceptualized that described how GPs approach and use guidelines in primary care: *learning to use guidelines by interactive contextualized dialogues, learning that establishes confidence to provide high quality care* and *learning by use of relevant evidence in the decision-making process.*

### Learning to use guidelines by interactive contextualized dialogues

The GPs emphasized that local collaboration and a learning dialogue were important to ensure that the patients would receive optimal care and an appropriate level of care. This was underpinned by the categories: “Feedback by peer-learning” and “Feedback by collaboration, mutual learning and equality between specialties”.

#### Feedback by peer-learning

The GPs stressed that recurrent structured group-dialogues at the PHC were not only a way of exchanging knowledge but were also stimulating, intellectually as well as socially, and offered an important opportunity for feedback. The GPs perceived that group-dialogues created power in decision-making and stimulated a feeling of community. The residents’ skills in information retrieval, combined with the considerable patient experience of the specialists in family medicine were interpreted as the main reasons for this. Learning obtained by interaction and thus encouraging reflection was perceived by the GPs to give a better quality of care.

“…these (dialogues), were introduced in a very structured way at our PHC. The idea was to ask “What should I do when I have diagnosed a patient with hypertension?” This was the first case we had and we thought “Oh, this is going to be so boring” It was not boring at all, in fact it was very exciting because we circulated and then everyone got to talk, not only the people that always talk, like me for example. There was suddenly time for reflection which we never have otherwise” – Female

#### Feedback by collaboration, mutual learning and equality between specialties

The GPs expressed pride in being residents or specialists in family medicine. They emphasized their important role, with overall responsibility for the patients, who often had multiple health problems. They also stressed that GPs could never refuse to take care of a patient. Practice guidelines were important when striving to give or to guide all patients to optimal care. Collaboration with secondary care in many specialties regarding patients with multiple illnesses was part of the daily practice for the GPs. Therefore, they emphasized the value of written practice guidelines that invited individual interpretations which depended on the clinical situation.

Practice guidelines written as strict detailed instructions with demands for specified investigations to be performed before consulting secondary care gave the GPs the feeling of being controlled, with little opportunity to influence patient care. Thus, it made them feel disrespected in regard to their competence as specialists in family medicine. This feeling of lack of understanding and lack of respect for the working situation in primary care caused frustration and irritation among the GPs, which was expressed during the interviews.

“Secondary care probably does not realize how we work. They might not realise that we are very busy. They think that they can refer everything to primary care… I handle the same kinds of patients now as a GP as I did when I used to work in internal medicine in secondary care, but today I am also a health coach and a secretary” – Female

In addition to this, GPs that were residents in family medicine were often instructed by their supervisors to strictly follow special flow-charts to optimize the care of the patients for financial reasons. The residents feared that this would lead to a reduced possibility to reflect on their decisions and thus would reduce their chance of learning from their own experiences.

The GPs suggested a more developed cooperation between primary and secondary care when designing the guidelines. This could be a way to make the practice guidelines more supportive and open to individualization of decisions, depending on the clinical situation. The GPs pointed out that to introduce new guidelines gradually and to evaluate them continuously would give feedback to secondary care on how the guidelines work. They believed that an improved dialogue between primary and secondary care would be beneficial in establishing mutual respect between the disciplines.

“When the guidelines were to be updated all the GPs were invited to a meeting…They presented the guidelines and explained why they had updated them, how we should handle the patients, when to refer them to secondary care and planned follow-up. It was a short presentation followed by a long dialogue…” Female

### Learning that establishes confidence to provide high quality care

In general the GPs perceived that the practice guidelines were a support to achieve knowledge to provide high quality care. This was reinforced by the following categories: “Confidence by confirmation”, “Confidence by reliability” and “Confidence by evaluation of own results”.

#### Confidence by confirmation

The GPs emphasized that one of the most important aspects of the usability of practice guidelines was that confidence in the content of guidelines gave a sense of control. They perceived that printed or internet-based practice guidelines had an important role as a necessary quality-check as well as a confirmation of their own knowledge. In general, residents wanted to compare different kinds of practice guidelines but they also combined short summaries with reading of more in-depth literature. However, the GPs that were specialists in family medicine and thus more experienced, relied on their own material gathered in easily accessible files, such as quick reference guides, articles from journals and notes from lectures.

“ … it gives me support that this is not something that I have made up, that this is the way to treat…”-Female

The GPs wanted the opportunity to self-assess their own knowledge by regular short tests regarding updated practice guidelines. Many of the GPs perceived this form of interactive learning with immediate feedback as a stimulating way to confirm and to achieve knowledge that would be usable in decision-making.

“…some newspapers have recurrent quizzes regarding the latest news. That could be something for the scientific journals or VISS (local web-based practice guidelines) regarding the updated guidelines… If you get 10 out of 10 correct answers you are probably quite updated, you can do a quiz once a month… they are performed rather quickly and it is… a better way of learning because you react when you gave the wrong answer and then can see the correct answer.”- Male

The GPs emphasized the increasing demands for efficiency in primary care as a stress factor. It hindered the optimal use of practice guidelines. In general, the GPs underlined that practice guidelines provide the possibility to be prepared in advance, to seek or to confirm one’s own pre-understanding before the patient visit. Further, they can give a feeling of more thorough knowledge, a sense of control, and thus confidence. However, when pressed for time the GPs either quickly checked practice guidelines they were already familiar with or made the decisions they thought were right without reading the guidelines. These strategies created feelings of anxiety and uncertainty, especially if they had experienced situations when these strategies had resulted in wrong decisions.

“…After a couple of years I felt that –” This goes like clockwork, I know how to do this” - and continued to work without checking the guidelines. And then suddenly something went wrong. And then I got scared and started to check again.” – Male

The GPs stressed that practice guidelines could be used not only to confirm their own knowledge but also to increase the confidence of the patient. Being able to confirm that the investigations and/or treatment were evidence-based gave the potential to increase the patient’s motivation and knowledge.

“… I can actually prove to the patient that nowadays it is no longer necessary to always treat otitis with antibiotics, that it isn’t something that I have made up.”- Female

#### Confidence by reliability

The GPs pointed out the importance of feeling trust and feeling that the source of knowledge was reliable. This was regardless of whether knowledge was mediated by using printed or internet-based practice guidelines, attending a lecture, asking a colleague or by conducting a formal consultation. A consultant, even if highly specialized in the area, was not perceived as reliable if he or she seemed to be pressed for time. The GPs perceived the greatest reliability from consulting someone they knew personally, regardless of whether it was a specialist at the hospital, a colleague in the PHC or a lecturer.

“I used to work in Gotland so I usually call the hospital there since I know everyone. There is never a resident physician answering the phone. So I am always familiar with the person that I am talking to.” – Female

A prerequisite for the GPs to feel confidence in using printed or internet-based practice guidelines was that the source was well known and that the guidelines were continuously updated. Internet-based practice guidelines directly linked to in-depth literature increased the sense of reliability and confidence. A suggestion from the GPs was that diagnoses in the internet-based guidelines should automatically be linked to safety aspects that could be formalized as: “Important factors to consider at the diagnosis X”. Safety aspects for the patients but also legal aspects for the GPs made the GPs prefer written answers to referrals and consultations, and these should be documented directly in the patient record if possible. Documented answers were thus preferred instead of phone-consultations if the GPs were not familiar with or did not feel confident with the consultant.

#### Confidence by evaluation of own results

The GPs perceived that the availability of easily accessible specialists to ask, either by telephone or, as some of the GPs had experience of, working in the same building, was optimal for obtaining a high quality of care. This creation of professional networks strengthened the feeling of autonomy and increased the curiosity and motivation of the GPs, and thus gave them greater confidence to handle patients with more complicated diseases. The GPs wished for individualized follow-ups with secondary care how the use of practice guidelines improved the care of their patients. They expressed the possibility that evaluation of individualized results in an audit-like model would be stimulating in the daily practice and would strengthen confidence in the practice guidelines.

“I would like to see results. I think that one reason for not following the guidelines is that we never have follow-ups. I want follow-ups, personal follow-ups. We hardly ever get feedback on what we do. The client healthcare organization has organized follow-ups which I think is good because then you get feedback. If I never get feedback on what I am doing…why should I care??”- Female

### Learning by use of relevant evidence in the decision-making process

The GPs perceived that the problem in using practice guidelines was not to get information but to find relevant information from the vast amount available. This was supported by the categories “Design and lay-out visualizing the evidence” and “Accessibility adapted to the clinical decision-making process”.

#### Design and lay-out visualizing the evidence

A prerequisite for the GPs to use the guidelines was that they had to be short, concise, with a clear overview and a pedagogic layout. They could be presented as a quick reference guide, in tables or as treatment cards with links or references to in-depth literature to consider later if needed. To quickly get an overview of the information gave the GPs a sense of control and thus reduced the feeling of stress.

“… it should not be too compact, there should be space in the document and the guidelines should be arranged in a recognizable pattern so that the information is easy to find.”- Female

Many guidelines today are sorted by diagnoses, which works well in many cases. However, some GPs felt that sorting practice guidelines by symptoms would be more appropriate for decision-making in primary care.

Lectures, especially in smaller groups, were perceived as a valuable learning activity by the GPs. All the GPs pointed out that a prerequisite for having lectures in the day-time was that they should take place at the local PHC. Having to leave the workplace for lectures was time-consuming and thus not cost-effective.

None of the GPs preferred practice guidelines presented as textbooks containing a lot of facts, not even as dictionaries. They were perceived as difficult to scan quickly, not suited to clinical practice and thus only used by GPs with special interest in the area. The GPs pointed out that practice guidelines not adapted to clinical practice were simply not used.

“One has to do a cost benefit analysis… I looked at these regional guidelines for children with asthma in Stockholm …that is 160 pages. It is too much if you just have simple questions regarding investigations and referral and it is too extensive to browse or skim through. It does not work in the context of primary care; short concise sources work better.”- Male

#### Accessibility adapted to the clinical decision-making process

Even if internet-based practice guidelines are the most common today, most GPs still preferred short, printed, and yearly updated brochures since they are perceived as the most easily accessible, lying on the desk. In internet-based sources it was often perceived as difficult to find the information required from among all the information given. Slow electronic systems also made the GPs feel frustrated.

“I feel that the biggest problem with the internet is lack of time. I lose control of time when I am searching for information on the internet and then I feel stressed having to keep track of time. “Oh God, there is so much information, where should I start”…” – Female

Besides computer systems more adapted to clinical practice the GPs wanted education to improve their information retrieval skills. The GPs believed that if they had improved skills in searching for information, they would perceive internet-based information as more accessible and thus would use it more. All the GPs perceived that links to detailed literature in the internet-based guidelines would enhance patient care and also save time.

Phone consultations were, to many of the GPs only accessible during certain times of the day and were therefore not used. However, GPs that had free access to phone consultations perceived them as the most valuable form of guidance in difficult cases.

Practice guidelines in many specialties are collected on the local website “VISS”, and the GPs appreciated having one, easily accessible site for the county council, where the local guidelines regarding treatment and level of care were clearly stated. However, the fact that the GPs perceived that VISS did not cover more uncommon diseases, was not always updated, and did not have links to more in-depth information caused frustration and sometimes uncertainty among the GPs and thus made VISS less usable. The GPs emphasized that secondary care should give priority to one site such as VISS when developing practice guidelines. This site should include more information on uncommon diseases, links to in-depth literature, and should describe the principles of referral of patients. Keeping the guidelines on this site continuously updated would further enhance their usefulness.

## Discussion

The possibility for learning to use guidelines by interactive contextualized dialogues and learning that establishes confidence in providing high quality care was emphasized by the participating GPs as important aspects to consider in their approach to the practice guidelines. A prerequisite for using the guidelines was that they should allow access to relevant evidence in the decision-making process.

Making correct clinical decisions for each individual patient is a central goal for all physicians. The findings in our study have highlighted the importance of the usability of the guidelines in the decision-making process. The themes conceptualized in this study could be linked in different ways to the Dual Process Theory [11-13].

In the first theme “Learning to use guidelines by interactive contextualized dialogues” the main finding was the importance of dialogue in decision-making in clinical practice. Pattern recognition is central in this dialogue. Regardless of whether the dialogue is obtained by peer-learning or by collaboration between primary and secondary care, it enhances the repetitive operation of the system 2 process and thus increases the process of pattern recognition. The opposite, strictly detailed instructions or flowcharts with few possibilities for personal reflection instead lead to the system 2 process overriding system 1, which will hinder the probability of pattern recognition.

The importance of networks in decision-making has also been pointed out by Mascia et al. who showed that physicians’ attitude towards evidence-based medicine (EBM) is strongly correlated with professional networks [19]. Their conclusion was to avoid marginalization and stimulate integration and continuity of care both within and across the boundaries between different healthcare providers. The importance of dialogue in decision-making has been emphasised by Norman in a review article [20]. He concluded that there is no single best way to solve a problem in the decision-making process. The components of knowledge and skills required to achieve the goal of effective care are complex and multidimensional. Feedback or dialogue is essential for this process. Another aspect of peer-learning by dialogue was that group discussions of patients with rare diseases could improve the knowledge as well as decrease the risk of diagnostic errors for GPs [21]. Practice guidelines designed as rigid, strictly detailed information were in our study perceived as assignments instead of being part of a continuous learning process since they did not offer any possibilities for personal reflections or dialogue. Thus, they were considered as barriers instead of facilitators in the decision-making process. These findings are in line with several other studies discussing barriers to following clinical practice guidelines [2,4,22].

In the second theme, “Learning that establishes confidence to perform a high quality of care”, the main finding was the importance of the GPs feeling confidence in the practice guidelines and having the possibility for evaluation of their decisions during the diagnostic process. The decision-making process is a balance between systems 1 and 2 that involves earlier experience and context on one side, and the use of practice guidelines, consultations or evaluation of one’s own results on the other side.

Confidence could be obtained by confirmation of knowledge, preferably by immediate feedback, but only if the source was perceived as reliable. Lack of confidence could result in the system 1 process overriding system 2, a dysrational override that would increase the risk of diagnostic errors.

Thus, if the GPs felt more confident by having the possibility to evaluate their own results they could switch between system 1 and 2 as needed. This would, as well as facilitating decision-making and decreasing the risk of diagnostic errors, also strengthen the autonomy of the GPs. To get feedback and to feel confident in competence would strengthen intrinsic motivation and thus autonomy. This, in turn, would promote behavioural changes as well as a positive attitude towards the use of the guidelines [23].

In the third theme, “Learning by use of relevant evidence in the decision-making process”, the main finding was that the most important aspect of using practice guidelines was to find relevant information. If the practice guidelines are easily accessible with a clear design and lay-out, the use of the system 2 process will be encouraged. This, in turn, will make it easier for the GPs to switch between system 1 and 2 during decision-making. If the practice guidelines do not fulfil these criteria there is a risk that the GPs will make their decisions based on their own experiences without considering the practice guidelines due to lack of time. This insufficient use of the type 2 system would, in turn, mean an increased risk of diagnostic errors in the decision-making process. This theory is supported by the study of Duran-Nelson et al. [24] where speed, trust and portability were identified as the biggest drivers for resource selection, while information overload appeared to be one of the biggest barriers. Previous studies have also pointed out guideline format as an important factor affecting whether GPs use guidelines or not. These studies concluded that practice guidelines should be short, simple and preferably should include patient leaflets [25,26].

We found that the “cognitive miser” strategy was necessary for the GPs in their daily routine work. The challenge when using the “cognitive miser” strategy is to keep a balance in the use of cognitive effort. Too little cognitive effort leads to high efficiency but a higher risk of diagnostic errors, while too much cognitive effort leads to a low risk of diagnostic errors but would probably not be compatible with the time constraints in clinical practice. GPs need to have relevant information at hand to be able to make a decision. Yet, they must have some pre-understanding to know which diagnoses to look for in the practice guidelines. Otherwise the “cognitive miser” strategy will result in the system 1 process overriding system 2. An example of how the “cognitive miser” strategy can lead to diagnostic errors is described in the study of Schmidt et al. [27]. In this study he showed a substantial availability bias causing a significant higher rate of diagnostic errors among experienced physicians compared to a control-group after exposure to media-provided information about a disease. In the next phase of the study when the exposure was the same but the physicians were given instructions (guidelines) and time for reflection there was no significant difference in diagnostic errors between the groups.

Previous studies [28,29] have shown that diagnostic errors are common. Most common were diagnostic reasoning errors when the physicians were not aware that their actions were incorrect. The conclusion was that better understanding of the decision-making process could help to improve the process and thus improve patient care. We think that it is important to consider the “cognitive miser” strategy when practice guidelines are elaborated and designed. Increasing the usability of the guidelines could thus reduce the risk of diagnostic errors.

There are several studies, mostly quantitative but also qualitative, exploring mainly the barriers but also the facilitating aspects of following practice guidelines [2,4,5,7,8,22]. In our study all participants worked at group practices, which may have influenced the results. The influence of the size of the primary health care centre in the decision-making process has been investigated in a systematic review by Damiani et al [30]. This review showed strong evidence that group practice performed better drug prescription according to the guidelines than single-handed practice. Further there was moderate evidence that GPs working in a group practice were more satisfied with their compensation and that group practice improved the utilization of information technology. The findings in this review are in line with our findings regarding the importance of dialogue and feedback in the learning process, which is easier to obtain in a group practice. Further our findings that accessibility to practice guidelines is a prerequisite for use are well in line with the findings in the review where group practice improved the utilization of information technology. Their conclusion was that there seems to be organizational advantages in group practice compared to single-handed practices in using the practice guidelines in the decision-making process. In our study the number of GPs varied between 6-19 GPs. Further studies are needed to investigate the optimal size of a group practice to obtain high quality of care.

Decision-making is central for a physician in clinical practice. The strength of this study is therefore that it focuses on exploring how GPs approach, learn from and use practice guidelines in their decision-making process, and discusses the results in the light of the Dual Process Theory.

However, given the design of the study, interviews can only give data regarding what the GPs say they do; not what they actually do in practice. Nevertheless, a relationship could be anticipated between what people say and do. One way to increase the validity between “saying and doing” is to ask questions that reflect the participants’ way of acting [31]. In our interviews we frequently used follow-up questions where we asked for concrete examples. The construction of the groups and the settings was initially planned to be equal in all four focus group interviews but this did not work out for practical reasons. Nevertheless, in three of the interviews there were lively discussions in which all the GPs participated equally. In the remaining interview the energy in the discussions was low and it was difficult to motivate two of the participants to speak. This lack of engagement could have been associated with the fact that this was the interview where the participants had the widest range of experience as physicians in primary care.

To provide optimal healthcare it is necessary that we understand how clinical evidence can be effectively transferred and applied in the context of primary care, and thus appreciate the importance of effective continuing education or professional development of physicians [32]. We have identified learning strategies that could be considered when developing and implementing new practice guidelines. The most important issue in CPD is to take the context into account, to arrange the learning activities to facilitate interaction between healthcare professionals, and finally to include feedback on performance based on guidelines and other knowledge sources. Regular meetings when GPs discuss pros and cons with different guidelines in primary care have a potential to facilitate this contextualization and interaction. Further concerned GPs would preferably be involved in the process of developing new practice guidelines aimed for use in primary care setting.

## Conclusion

Decision-making is central for GPs in their day-to-day practice. It is a dual process that involves using intuitive and analytic thinking in a balanced way in order to reduce the risk of diagnostic errors and improve the quality of care. We have identified key aspects of learning in attitude towards and use of practice guidelines, thereby improving quality in the decision-making process. We hypothesize that learning based on evidence-based practice should start from the physicians ‘own experiences and the special circumstances of the primary care context. It should include activating learning methods that foster dialogue and reflective capacity in order to improve evidence-based decision-making. Easy access to short guidelines perceived as trustworthy, and the opportunity to get feedback on one’s own results were also important for ensuring high quality in the decision-making process. To learn with colleagues provides opportunities to discuss and solve local organizational problems in accordance with the practice guidelines.

Future studies are needed to further increase the understanding of effective learning strategies to routinely use evidence-based medicine in the clinical decision-making process.

## Competing interests

The authors declare that they have no competing interests.

## Authors’ contributions

MI, PBR and AK participated in the design of the study, analysed the data and drafted the manuscript. MI collected the data and performed the interviews together with PBR. All the authors read and approved the final manuscript.

## Pre-publication history

The pre-publication history for this paper can be accessed here:

http://www.biomedcentral.com/1471-2296/15/141/prepub
